# Editorial: Insight in non-pharmacological treatment of pain—2023

**DOI:** 10.3389/fpain.2024.1512987

**Published:** 2024-12-04

**Authors:** Geoffrey Dover

**Affiliations:** ^1^ Department of Health, Kinesiology, and Applied Physiology, Concordia University, Montreal, QC, Canada; ^2^ CRIR - Centre de Réadaptation Constance-Lethbridge du CIUSSS COMLT, CIUSSS West-Central Montreal, Montreal, QC, Canada

**Keywords:** pain catastrophizing, fracture, pain self-efficacy, non-pharmacological treatment, kinesiotape

**Editorial on the Research Topic**
Insight in non-pharmacological treatment of pain—2023

I still remember my first challenging experience assessing pain in a clinical environment. I was working at a rural sports camp in northern Ontario. There were about 400 new athletes each week that competed in various sports. One day, there was a 12-year-old male basketball player that fell on an outstretched hand. By the time I got to him, he was sitting on the ground holding his arm. Upon inspection, there was a deformity at the distal radius that looked like he had two wrist joints. I asked him if he was OK, and he said he was. He looked so calm for something that looked very painful, which made the whole scenario a bit confusing. I asked him if he was in pain and he calmly said “yes” and just continued to look at me. I was puzzled by his relaxed demeanour, so I asked again if he was OK and he said yes but that his wrist just hurt a lot. So I started to splint the injury and told him we will have to take him to the hospital. He said OK and calmly let me finish the splint. Sometimes in this case we would call an ambulance, but since he said he was OK several times, we just got one of the counselors to drive him to the hospital. For some reason when they were leaving I asked him to get a copy of the x-ray and bring it back with him. Since I recently earned my bachelors degree I guess I felt like I was a burgeoning radiologist.

Then about a week later there was a different 12-year-old boy playing basketball. He too fell on an outstretched hand and injured himself. When I walked over, he was screaming and in a lot of pain. I had a hard time just getting to look at his wrist since he was writhing around so much. When I was able to expose the lesion site, he had a deformity at the distal radius and it looked like he had two wrist joints. I was asking him some other questions but he was in so much pain he could not answer me and he was very pale and seemed to be going in and out of consciousness. We had to call an ambulance to take him to the hospital and again I asked for a copy of the x-ray.

Both kids had to leave the camp early, but I was left with the x-ray from both individuals. When I put them side by side I was struck by how identical the fractures were. Two colles fractures: the distal part of the radius fractured in the same place and in the same fashion in both wrists. Yet, one person was in a little amount of pain and one person was in a horrendous amount of pain. Since I recently graduated and was taught the biomedical model of pain, I could not understand why one person experienced so much more pain compared to the other, considering the fractures were nearly identical on the x-ray and the amount of soft issue damage appeared the same during the assessment. If the lesion sites were so similar, how was one generating such a higher pain response compared to the other?

Little did I know at the time that was the first pain experience that really led me to study pain for the next several years and how prevalent pain and pain research would become. Pain is something that is a problem worldwide (1). There are thousands of researchers, and clinicians examining pain, and trying to come up with the best solutions for treating pain using the best possible evidenced based approach. As a result of all these studies on pain, researchers have identified several factors that can influence the amount of pain someone experiences including but not limited to: depression, fear avoidance, pain catastrophizing, pain self-efficacy, and others (2).

There are numerous factors that can influence pain, and these factors may contribute to the reason why medication is less effective for treating pain and supports the need for other comprehensive non-pharmacological treatments which is the topic of this special issue. There is evidence that opioids are less effective for treating pain and have an increase in risk (3), which has led others to call for more non-pharmacological treatments (4). There is a lot of research on pain now, which was evident when putting together this special issue which includes two reviews where authors attempt to consolidate research into guidelines that can help shape clinical treatment. The review by Wolfe et al. examined the effect of various electrical modalities on low back pain and other patient outcomes including psychosocial measures. The review by Foy et al. evaluated studies that used psychological and behavioral interventions to treat pain and pain related outcomes. In addition, another article in this special issue tackled the challenge of examining the mechanism for pain. Medina et al. used functional MRI to measure brain functional connectivity in people suffering from fibromyalgia. These authors noted an improvement in pain catastrophizing in people receiving a mindfulness or an educational program. Moreover, they noted that changes in salience and sensorimotor networks have the potential to predict mind-body response treatment. Both reviews and the experimental study included measures of psychosocial factors which can help explain why some people feel more pain than others. The hard part is turning research results into a change in clinical practice since some suggest that it takes 15–20 years for research to be adopted to clinical applications (5). Special issues like this can also share findings from a variety of interventions to help clinical practice. Our special issue includes two articles that indicated kinesiotaping and integrated naturopathy and yoga could improve painful conditions (Tudini et al. and Paudel et al. respectively). Our goal as clinicians and researchers is to continue to improve our interventions and individualized treatment for those who have suffered a significant injury and experience a high level of pain, as well as those who don't.
